# Spatio-temporal patterns of dengue in Bangladesh during 2019 to 2023: Implications for targeted control strategies

**DOI:** 10.1371/journal.pntd.0012503

**Published:** 2024-09-20

**Authors:** Kamal Hossain, Sukanta Chowdhury, Ireen Sultana Shanta, Mohammad Sharif Hossain, Probir Kumar Ghosh, Mohammad Shafiul Alam

**Affiliations:** Infectious Disease Division, International Centre for Diarrhoeal Disease Research, Bangladesh (icddr,b), Mohakhali, Dhaka, Bangladesh; McGill University Faculty of Medicine and Health Sciences, CANADA

## Abstract

**Background:**

Dengue, a viral infection transmitted by *Aedes* species mosquitoes, presents a substantial global public health concern, particularly in tropical regions. In Bangladesh, where dengue prevalence is noteworthy, accurately mapping the distribution of high-risk and low-risk areas and comprehending the clustering of dengue cases throughout the year is essential for the development of effective risk-based prevention and control strategies. Our objective was to identify dengue hotspots and temporal patterns over the years across Bangladesh in the years 2019–2023 excluding year 2020.

**Methods:**

A sequential spatial analysis was employed for each year to identify high-risk areas for dengue cases. Choropleth graphs were used to visualize the geographic distribution of dengue incidence rates per million population across the areas. Monthly distribution analysis was performed to identify temporal trends over the year 2022 and 2023. Additionally, the global Moran’s I test was used to assess the overall geographical pattern. Subsequently, Anselin local Moran’s I test was employed to identified clustering and hotspots of dengue incidences.

**Results:**

Dengue cases in Bangladesh exhibited a significant increase from 2019 to 2023 (excluding 2020 data), with a cumulative total of 513,344 reported cases. Dhaka city initially bore substantial burden, accounting for over half (51%) of the 101,354 cases in 2019. The case fatality rate also demonstrated a steadily rise, reaching 0.5% in 2023 with 321,179 cases (a five-fold increase compare to 2022). Interestingly, the proportion of cases in Dhaka city decreased from 51% in 2019 to 34% in 2023. Notably, the southeast and central regions of Bangladesh showed the highest dengue rates, persisting throughout the study period. Cases were concentrated in urban regions, with Dhaka exhibiting the highest caseload in most years, followed by Manikganj in 2023. A distinct temporal shift in dengue transmission was observed in 2023, when the peak incidence occurred three months earlier in July with complete geographic coverage (all the 64 districts) compared to the peak in October 2022 (covering 95%, 61 districts). Positive global autocorrelation analysis revealed spatial dependence, with more stable trends in 2023 compared to previous years. Several districts like, Bagerhat, Barisal, and Faridpur remained persistent hotspots or emerged as new hotspots in 2023. Conversely, districts like Dinajpur, Gaibandha, Nilphamari, Rangpur and Sylhet consistently exhibited low caseloads, categorized as dengue coldspots throughout most of the years. Jhalokati in 2019 and Gopalganj in 2022, both initially classified as low-incidence district surrounded by high-incidence districts, emerged as hotspots in 2023.

**Conclusion:**

This study sheds light on the spatiotemporal dynamics of dengue transmission in Bangladesh, particularly by identifying hotspots and clustering patterns. These insights offer valuable information for designing and implementing targeted public health interventions and control strategies. Furthermore, the observed trends highlight the need for adaptable strategies to address the region’s evolving nature of dengue transmission effectively.

## Author summary

Dengue poses a serious global health threat, particularly in tropical regions like Bangladesh. Effective prevention and control depend on accurately mapping high-risk (hotspots) and low-risk (coldspots) areas. Using sequential spatial analysis, this study revealed a concerning rise in dengue cases, with a total of 513,344 reported of the study periods. The case fatality rate also increased, rising from 0.16% in 2019 to 0.5% in 2023. Initially, Dhaka city bore the highest burden, accounting for over half of the cases in 2019. However, a worrying shift emerged in 2023. Cases surged nationwide, with geographical coverage peaking in July–three months earlier than the typical October peak observed in 2022. This surge achieved complete geographic coverage, unlike the previous year. Spatial analysis indicated a strong spatial dependence of the disease, with trends stabilizing in 2023 compared to previous years. The study identified persistent hotspots in Bagerhat, Barisal, and Faridpur districts, while Dinajpur, Gaibandha, Nilphamari, Rangpur, and Sylhet districts emerged as coldspots. The southeast and central regions consistently showed high dengue rates. These findings underscore the dynamic nature of dengue transmission in Bangladesh, emphasizing the need for adaptable public health strategies and targeted interventions.

## Introduction

Dengue is a leading global health concern, caused by the dengue virus (DENV) and transmitted primarily by *Aedes aegypti* and *Aedes albopictus* mosquitoes [1,2]. With approximately half of the world’s population now residing in areas at risk, the annual estimate of dengue infections ranges from 100 to 400 million cases [3]. This viral illness thrives in tropical and subtropical climate regions, predominantly affecting urban and semi-urban regions globally [1,3]. While dengue poses a substantial challenge on a global scale, it holds a unique local significance as well. Bangladesh, high densely populated country in South Asia, has grappled with recurring dengue outbreaks in recent years, characterized by dynamic transmission patterns that burden its healthcare system in terms of increased mortality, patient overflow, difficult to accommodate patients, economic loss particularly in vulnerable communities [4].

Dengue outbreaks have posed a significant public health challenge in Bangladesh in recent years. Prior to the COVID-19 pandemic, the largest outbreak occurred in 2019, with over 100,000 reported cases, placing a substantial burden on healthcare systems after the first recorded outbreak in 2000 [5]. The subsequent years saw continued dengue activity, with outbreaks in 2020 (1,193 cases) and 2021 (28,429 cases) [5]. However, the true magnitude of these outbreaks might be underestimated due to the overlapping COVID-19 pandemic, which may have diverted resources and impacted case reporting [5]. This trend continued in 2022 with a similar number of cases (28,429), culminating in a massive outbreak in 2023 with a staggering 321,179 reported cases. A shift in the predominant circulating dengue serotype from DENV-3 (2019–2022) to DENV-2 in 2023 is believed to have contributed to the increase in hospitalization and case fatality rates [6]. This recent dengue outbreak presents a formidable threat to public health in Bangladesh, where healthcare resources are continually stretched to their limits due to both sporadic cases and periodic epidemics [5,7].

Effective dengue control strategies hinges on the identification of high-risk areas, “hotspots” where disease transmission is concentrated. Precise delineation of these hotspots facilitates targeted intervention strategies, resource allocation, and preventive measures [8,9]. Spatial analysis, incorporating cutting-edge geospatial technologies and advanced statistical techniques, has emerged as an invaluable tool in this pursuit. It offer valuable insights into the complex interplay between environmental factors, vector dynamics, and human population characteristics, ultimately aiding in the development of effective dengue control strategies [10].

Despite valuable insights gleaned from previous studies on the spatial distribution of dengue in Bangladesh, significant limitations and knowledge gaps persist. Most of the previous studies have focused on urban settings, particularly Dhaka city, and relatively small geographic areas [11–13]. This urban-centric approach overlook crucial dynamics in rural and peri-urban regions where distinct hotspots could be emerging [14]. Additionally, some studies have focused on comparing Dhaka city to other districts, rather than identifying distinct hotspots across the country [15]. Some studies have focused solely on the spatial distribution and hotspot of cases using limited data, without considering other factors like population, that contribute to dengue transmission dynamics [5,16]. However, a prior study evaluated spatio-temporal clusters of dengue in Bangladesh, using both cases and population data, but from 2000–2009 [17]. Dengue incidence in Bangladesh has exhibited a significant increase in recent years, with a wider geographic spread across the country. This necessitates a renewed evaluation of the spatiotemporal patterns of dengue to inform the development of targeted public health interventions.

Motivated by the limitations identified in prior research and the recent surge in dengue cases observed across Bangladesh, a comprehensive spatial analysis was conducted to identify dengue hotspots nationwide. The analysis encompassed the period from 2019 to 2023, excluding data for 2020 due to limitations. By pinpointing these high-risk areas and elucidating the spatial patterns exhibited by these hotspots, this study aimed to contribute valuable insights for the development of targeted and impactful public health interventions for dengue prevention and control.

## Methods

### Study Area and Population

Bangladesh, a densely populated country surpassing 165 million inhabitants, is located in South Asia. Its capital city, Dhaka, is home to approximately 14.7 million people [18,19]. Administratively and geographically, the country is divided into eight divisions further encompassing 64 districts. Bangladesh is classified as a lower middle-income country and is pursuing the status of a least developed nation [20]. The study sites included all eight divisions and 64 districts across Bangladesh. The study population consisted of all individuals residing in the selected districts during the study period.

### Data Sources

We gathered data from multiple sources including the Bangladesh government’s official website and the daily press release of the Ministry of Health and Family Welfare [21], which provided updated daily information on dengue cases, fatalities, and the district-wise distribution. This existing dengue surveillance systems collected data from a number of healthcare facilities, encompassing 12–20 public and 29–58 private hospitals within Dhaka and 1–2 major hospitals per district throughout the rest of the country from 2019 to 2023. We compiled dengue cases per district nationwide from January 1, 2019 to December 31, 2023 [21] and excluded the data for 2020 due to unavailable of districts wise data. We utilized the District-level population data from the recent 2022 census conducted by the Bangladesh Bureau of Statistics (BBS) [19]. Additionally, we used Spatial boundaries of Bangladeshi districts as shapefiles from the "bangladesh" package within the R statistical environment [22].

### Data analysis

We projected the population for 2023 with the population of 2022 and the growth rate, 1.22% and population for 2019 and 2021 with the annual adjusted growth rate, 1.37% [19]. We estimated the dengue incidence rate (DIR) per 1000,000 people for each district by establishing the ratio between the dengue case count and the respective district’s population of that year and multiplied by 1000,000.

**Fig 1:**
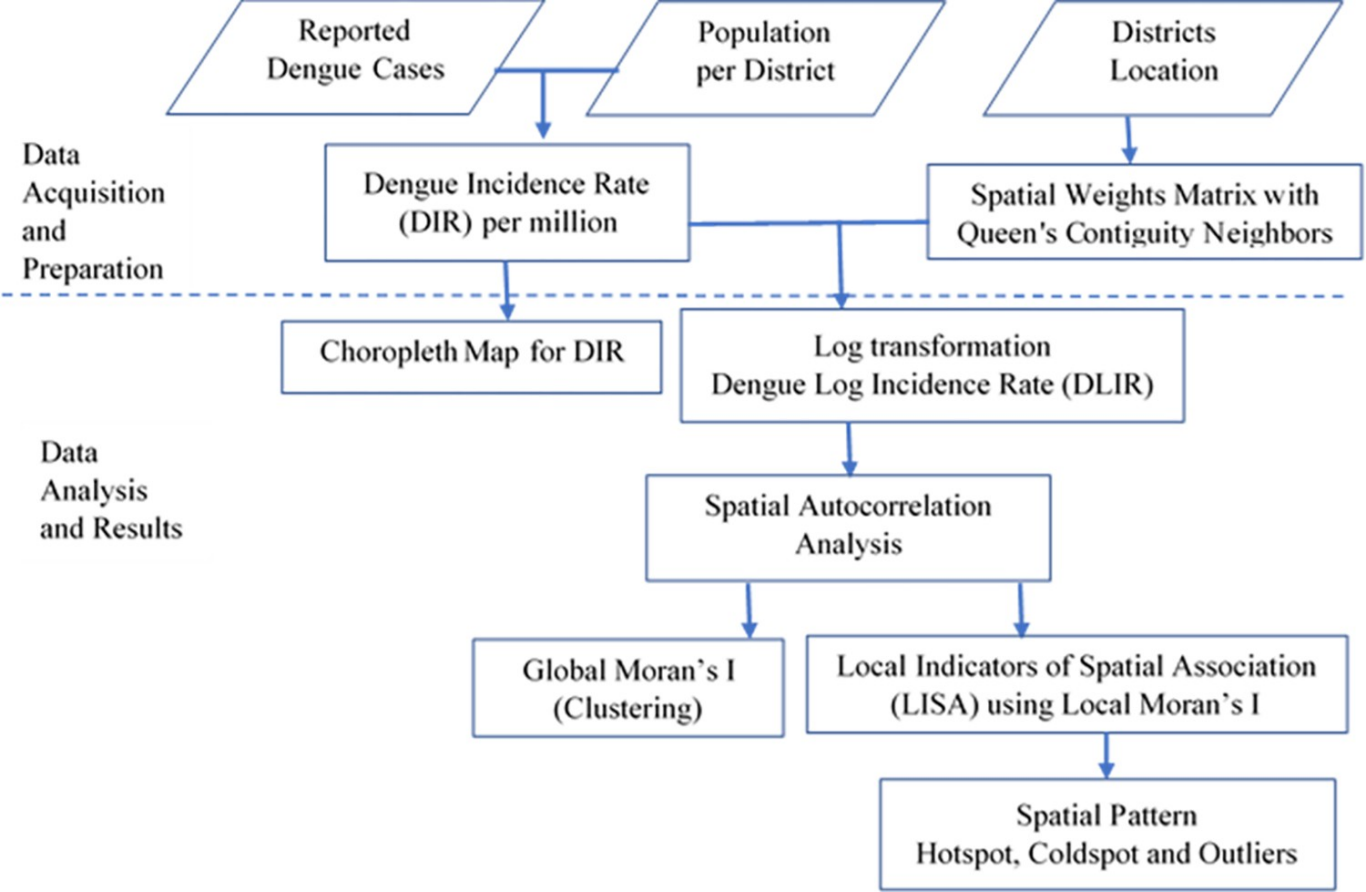
Flowchart of Methodology.

We conducted the spatial and temporal analysis of dengue incidence rate in Bangladesh’s districts in three phases (Fig 1). **First**, we georeferenced district boundaries and linked them to monthly/yearly dengue incidence rates. Subsequently, we generated choropleth maps with homogenous categories by Fisher-Jenks algorithm.

**In the second phase**, we investigated the existence of overall geographic clustering of dengue cases in the country for each year by using the global Moran’s I test. To establish spatial relationships among districts, we used dengue log incidence rate (DLIR) instead of incidence rate as DIR was non-normal distribution with heavily skewed to the right and created a spatial weight matrix. Neighboring districts were identified using first-order Queen’s contiguity, considering districts that shared borders or a common vertex as neighbors. We selected spatial lag one to characterize global clustering after examining spatial autocorrelation at higher-order spatial lags [23].

**In the third phase**, we employed local indicators of spatial analysis (LISA) [24] to pinpoint the districts with distinct spatial clustering patterns using the local Moran’s I statistic for dengue log incidence rate (DLIR). The LISA classification identified five distinct types of districts based on dengue incidence and spatial clustering patterns:

1. High-High (hotspot): Districts with high dengue incidence surrounded by other high-incidence districts (positive centralized DLIR and positive centralized lagged DLIR, significant local Moran’s I test, P<0.05).
2. Low-Low (coldspot): Districts with low dengue incidence surrounded by other low-incidence districts (negative centralized DLIR and negative lagged DLIR, significant local Moran’s I test, P<0.05).
3. Low-High (outlier): Districts with low dengue incidence surrounded by high-incidence districts (negative centralized DLIR, positive centralized lagged DLIR, significant local Moran’s I test, P<0.05).
4. High-Low (outlier): Districts with high dengue incidence surrounded by low-incidence districts (positive centralized DLIR, negative centralized lagged DLIR, significant local Moran’s I test, P<0.05).
5. Not Significant: Districts with no statistically significant spatial clustering of dengue (P> = 0.05).

Hotspots indicate locations facilitating dengue spread, needing targeted interventions. Coldspots offer research opportunities to understand lower rates, informing prevention strategies. Spatial outliers (high–low, low–high) hold epidemiological significance, requiring specific interventions for transmission risk reduction and protection in potential receptor areas.

We conducted all analyses and visualizations using R (version 4.2) [25] with the packages base, bangladesh, sf, sp, spdep, ggplot2, tmap [22,26–29].

## Results

The study identified a concerning increase in the case fatality rate alongside 513,344 dengue cases reported nationwide between January 1, 2019, and December 31, 2023 (excluding data from 2020).

In 2019, health officials reported a total of 101,354 dengue cases, with 164 deaths resulting in a case fatality rate of 0.16%. The capital city, Dhaka, emerged as the epicenter of the outbreak, bearing a substantial burden with over half (51,810; 51%) of the reported cases. While the number of cases dropped in 2021 to 28,429, the case fatality rate rose to 0.37% (105 deaths). Notably, over 83% of these cases (23,617) were again concentrated in Dhaka city. The year 2022 saw a resurgence in cases, with 62,382 reported, and a concerning rise in fatalities (281 deaths) pushing the case fatality rate to 0.45%. Although Dhaka city continued to be the epicenter, the proportion of cases reported there decreased slightly to 63%. However, 2023 stands out as the year with the most significant outbreak (cases more than five times than that of 2022). A total of 321,179 cases were reported, with a case fatality rate of 0.5%. While the proportion of cases in Dhaka city dropped further to around 34% (108,733).

### Dengue incidence

Dengue incidence rates exhibited marked variations between districts across the districts from 2019 to 2023. Dhaka emerged as the clear epicenter in 2019 with 3,772 cases per million people followed by Barisal and Jessore with 1,482 and 1,378 cases per million, respectively. In contrast, Sunamganj, Panchagarh, and Netrakona have seen minimal dengue incidence which was below 50 cases per million.

In 2021, the spatial distribution of dengue incidence remained similar, with Dhaka reporting the highest incidence at 1,626 cases per million. However, Gazipur and Patuakhali emerged as new areas with rates exceeding 150 cases per million. Interestingly, several districts, including Lalmonirhat, Noakhali, Panchagarh, Rangamati, Sunamganj, and Thakurgaon, had not reported any dengue cases.

Dengue incidence surge further in 2022, with Dhaka maintaining the highest burden of 2,675 cases per million populations. Notably, coastal districts like Cox’s Bazar and hilly district like Bandarban also emerged as areas of concern, exceeding 650 cases per million populations. Conversely, Gaibandha and Kurigram districts remained free of reported dengue cases, and Sunamganj continued to experience minimal burden with 2.6 cases per million populations.

The situation escalated dramatically in 2023 when Manikganj district became the new epicenter, grappling with a staggering 8,200 cases per million populations. Dhaka and Pirojpur also reported significant dengue burdens, exceeding 7,500 and 6,000 cases per million populations, respectively. Sunamganj again recorded as the lowest rate of 37 cases per million populations.

### Temporal and Spatial patterns of Dengue Incidence Rate

**Fig 2:**
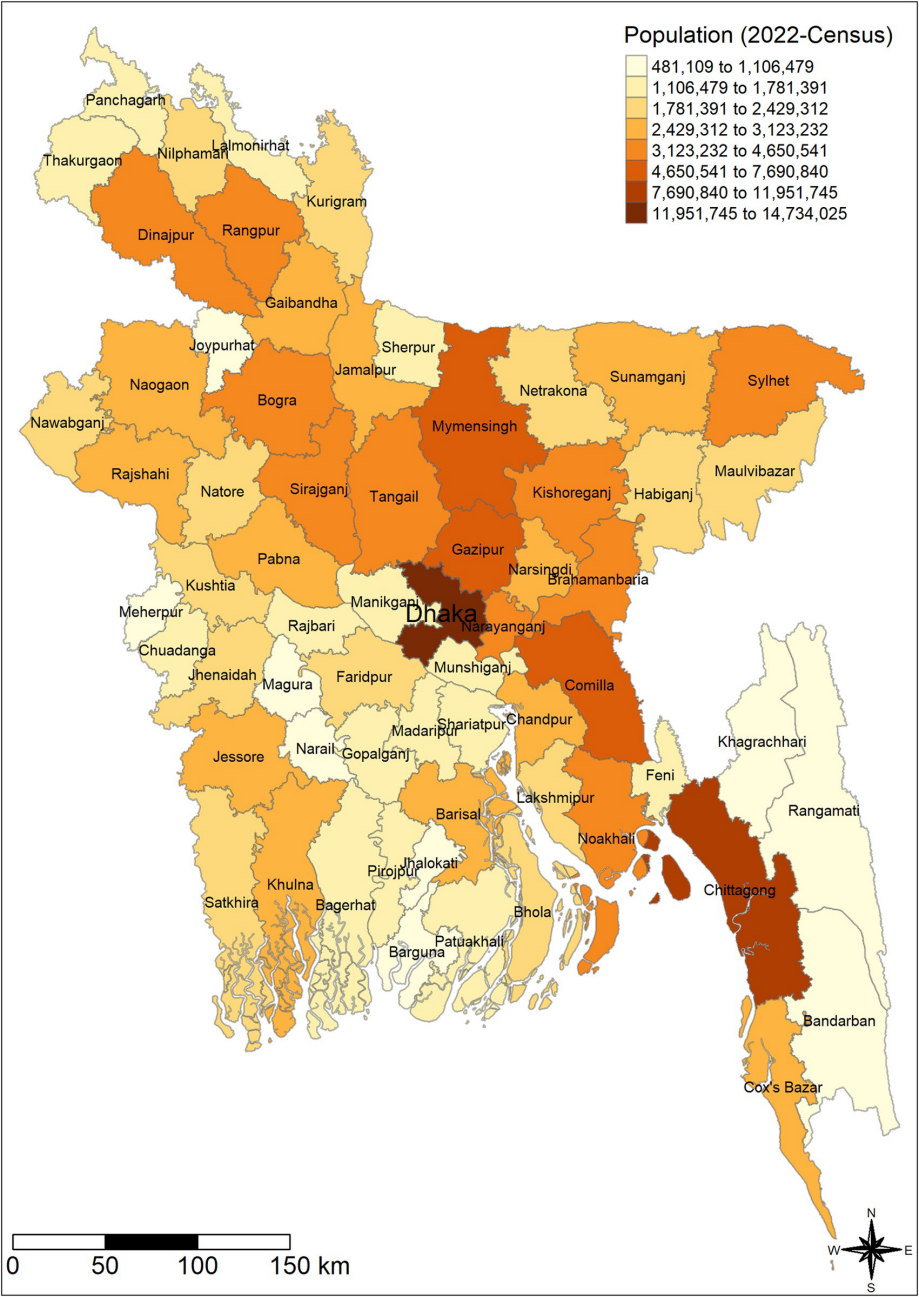
The spatial (districtwide) distribution of census population of Bangladesh in 2022. We have created the map using districts level shape file obtained from the R package named "bangladesh": https://cran.r-project.org/web/packages/bangladesh/index.html.

**Fig 3:**
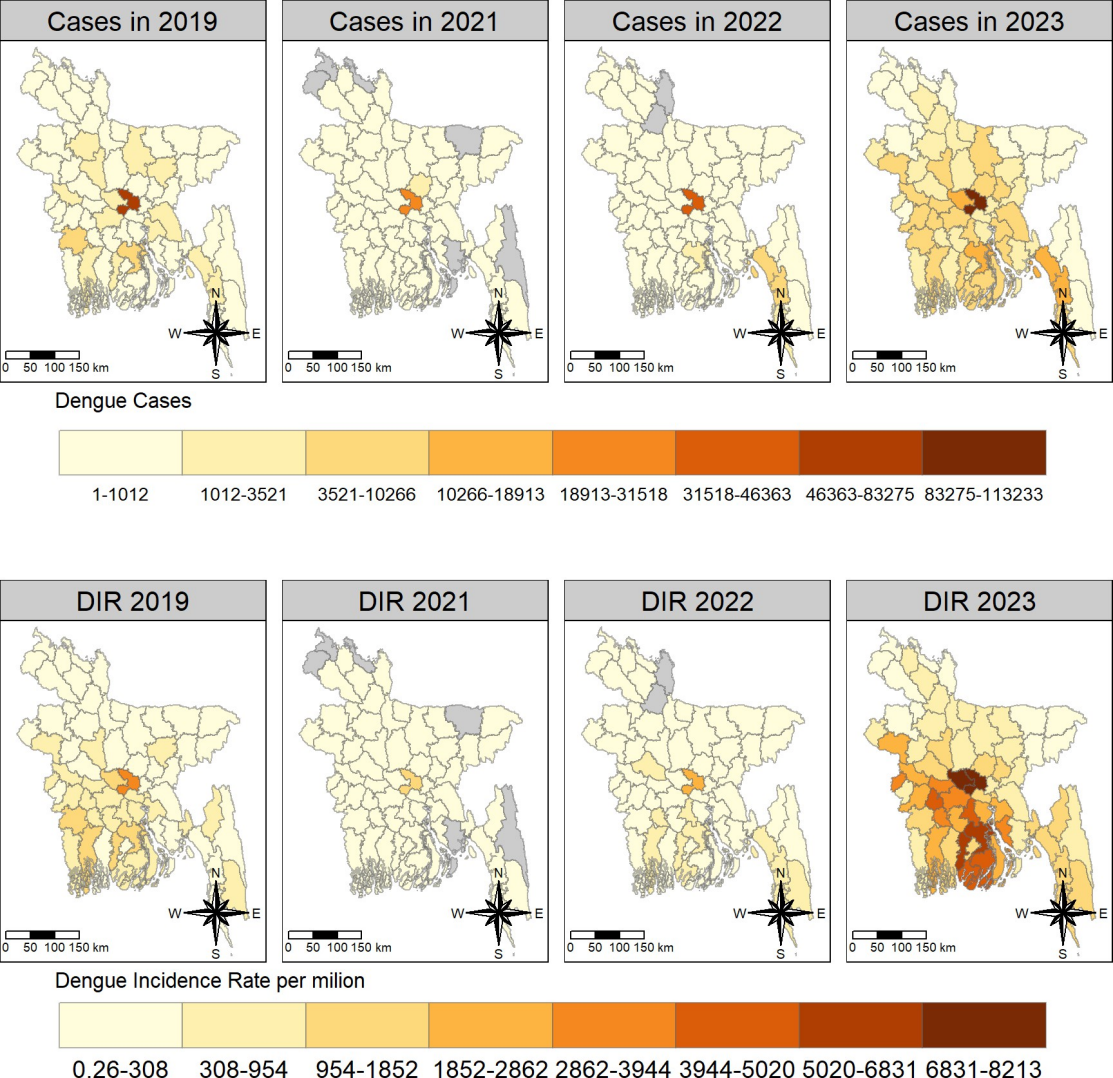
The spatial distribution of districtwide dengue cases and dengue incidence rate (DIR) per million in Bangladesh in 2019–2023. We have created all the maps using districts level shape file obtained from the R package named "bangladesh": https://cran.r-project.org/web/packages/bangladesh/index.html.

The choropleth graph exhibited a spatial pattern of population in 2022 and dengue incidence rate (DIR) across districts of Bangladesh over the years. The highest incidence rates per million were found in the southeast and central regions in 2019–2023 (Figs 2 and 3).

**Fig 4:**
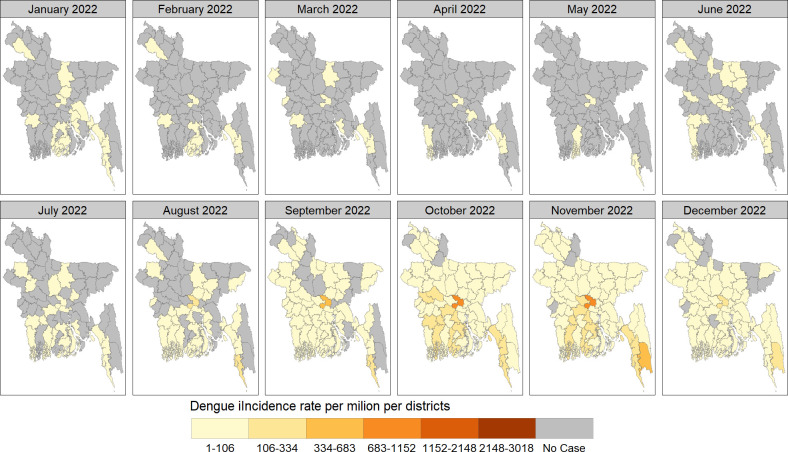
The spatial distribution of dengue incidence rate per million by month across districts of Bangladesh in 2022. We have created all the maps using districts level shape file obtained from the R package named "bangladesh": https://cran.r-project.org/web/packages/bangladesh/index.html.

**Fig 5:**
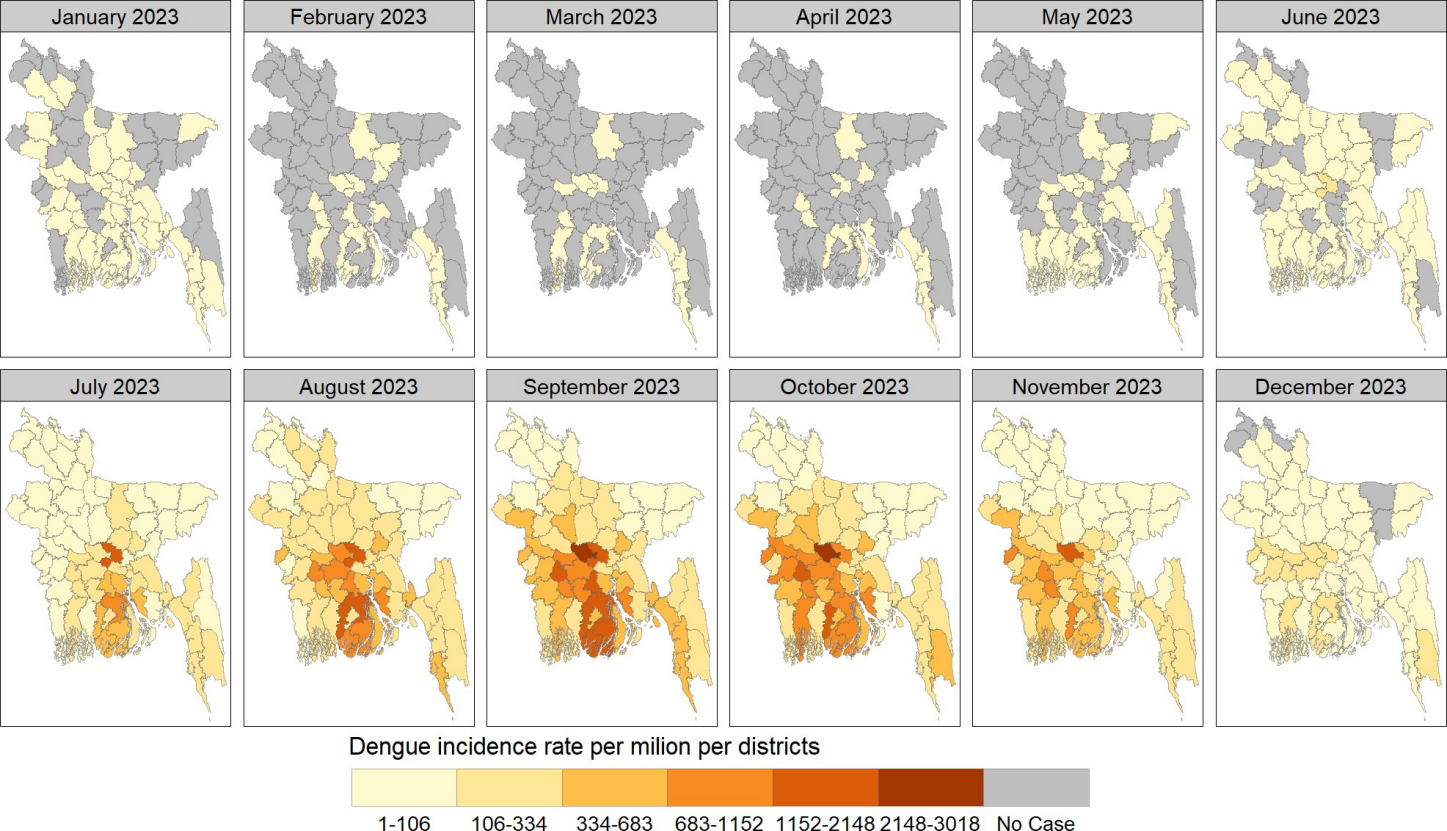
The spatial distribution of dengue incidence rate per million by month across districts of Bangladesh in 2023. We have created all the maps using districts level shape file obtained from the R package named "bangladesh": https://cran.r-project.org/web/packages/bangladesh/index.html.

**Fig 6:**
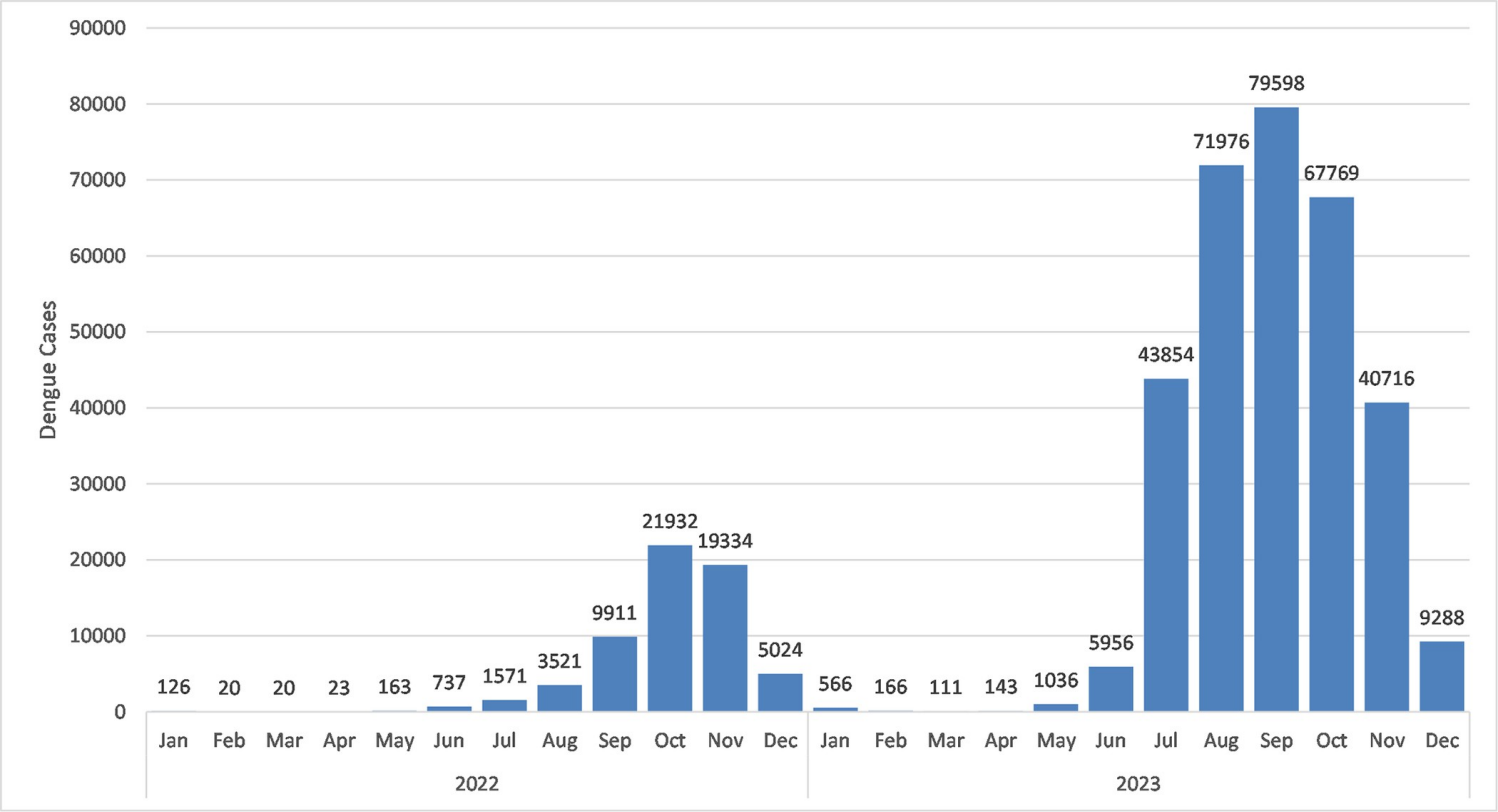
Monthly distribution of dengue cases across Bangladesh in 2022–2023.

The spatial distribution of monthly dengue cases per district in Bangladesh during 2022–2023, shown in Figs 4–6. Urban centers, particularly Dhaka, consistently reported the highest case concentrations. In January 2022, 15 districts (23%) were affected, reducing to 3–7 districts (5%-11%) in subsequent months. From June onwards, affected districts increased, peaking at 95% coverage (n = 61) in October and November, then declining to 54 (84%) in December, 2022. January 2023 saw widespread cases in 37 (58%) districts, decreasing to 11–16 districts (17%-25%) in the following months. May marked a rise, reaching 100% coverage (n = 64) in July and November 2023 and then declining to 59 (92%) districts in December, 2023

### Global spatial autocorrelation and clustering

We found constant significant (P<0.001) positive autocorrelation using the global Moran’s I test in each year across the country. The Moran’s I values were relatively stronger in 2023 (I = 0.66) than previous years. Notably, for districts further apart (higher-order adjacencies), the analysis exhibited negative autocorrelation or no evidence of spatial correlation, indicating dissimilar incidence rates between these districts (Table 1).

**Table 1:** Global Spatial Autocorrelation Analysis of dengue log incidence rate (DLIR) with higher order neighbor districts in the study periods.

	Dengue Log incidence rate (DLIR)				
	Global Moran’s I (P-value)				
**Years**	**1**^**st**^ **order**	**2**^**nd**^ **order**	**3**^**rd**^ **order**	**4**^**th**^ **order**	**5**^**th**^ **order**
2019	0.43 (P<0.01)	0.39 (P<0.01)	0.17 (P<0.01)	-0.02 (P = 0.56)	-0.24(P = 1)
2021	0.27 (P<0.01)	0.16 (P<0.01)	0.05 (p = 0.10)	0.02 (P = 0.19)	-0.16 (p = 1)
2022	0.52 (P<0.01)	0.33 (P<0.01)	0.10 (P = 0.02)	0.05 (P = 0.09)	-0.15 (P = 1)
2023	0.66 (P<0.01)	0.49 (P<0.01)	0.19 (P<0.01)	-0.03(P = 0.66)	-0.28 (P = 1)

### Local spatial autocorrelation and spatial pattern

**Fig 7:**
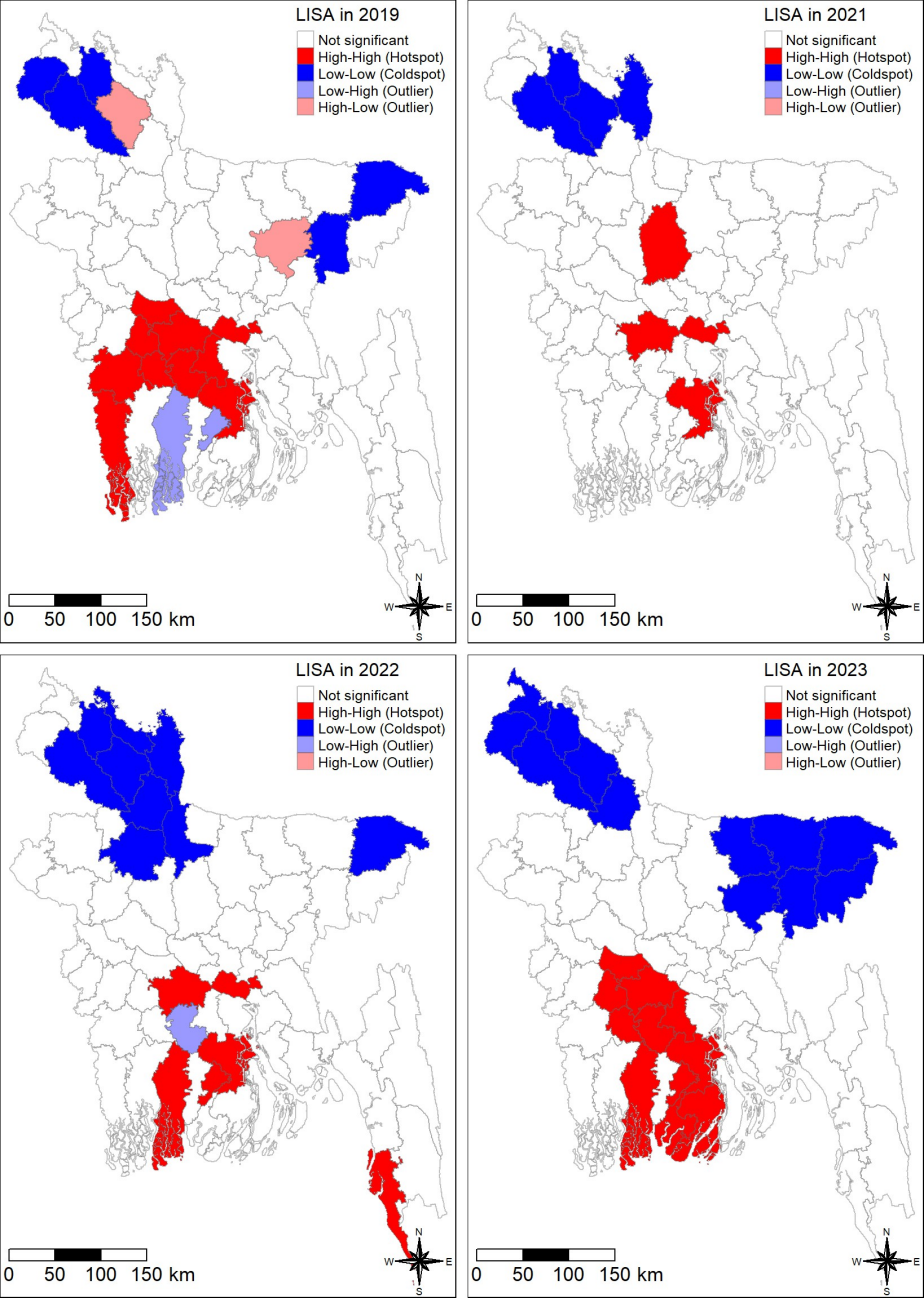
The spatial pattern of dengue including hotspot and coldspot in Bangladesh in 2019–2023. We have created all the maps using districts level shape file obtained from the R package named "bangladesh": https://cran.r-project.org/web/packages/bangladesh/index.html.

Using local Moran’s I statistics, we identified significant spatial clustering (P-value < 0.05) of dengue outbreaks across Bangladesh from 2019 to 2023.This analysis revealed a dynamic spatial distribution, characterized by distinct hotspots, coldspots, and spatial outliers (Fig 7).

The spatial analysis revealed persistent hotspots of dengue incidence in the southern and central regions of Bangladesh throughout the study period. In 2019, the hotspots were concentrated in Barisal, Faridpur, Gopalganj, Jessore, Madaripur, Magura, Munshiganj, Narail, Rajbari, and Satkhira. A contraction of hotspots area was observed in 2021, primarily concentration to Barisal, Faridpur, Munshiganj, and Tangail. However, the outbreak re-emerged in 2022 with Bagerhat, Cox’s Bazar, and Jhalokati emerging as new hotspots, alongside the persistent hotspots in Barisal, Faridpur, and Munshiganj. The year 2023 witnessed the most widespread outbreak, with hotspots encompassing Bagerhat, Barguna, Barisal, Faridpur, Gopalganj, Jhalokati, Madaripur, Magura, Narail, Patuakhali, and Rajbari (Fig 7).

Coldspots characterized by lower concentration of dengue incidence, were predominately located in the northern and northeastern regions of Bangladesh. In 2019, the coldspots included Dinajpur, Habiganj, Nilphamari, Sylhet, and Thakurgaon. This spatial pattern largely persisted in 2021, with Dinajpur, Kurigram, and Nilphamari remaining coldspots. An expantion of coldspots area was observed in 2022, encompassing Bogra, Dinajpur, Gaibandha, Jamalpur, Kurigram, Lalmonirhat, Nilphamari, Rangpur, and Sylhet. On the other hand, the coldspots in 2023 included Dinajpur, Gaibandha, Habiganj, Kishoreganj, Maulvibazar, Netrakona, Nilphamari, Panchagarh, Rangpur, Sunamganj, Sylhet, and Thakurgaon districts (Fig 7).

The spatial analysis additionally revealed the presence of Spatial outliers throughout the study period. These outliers represent districts with dengue incidence concentrations (either high or low) that deviated significantly from surrounding areas. In 2019, low-high outliers (districts with low case concentrations surrounded by high case concentrations) were identified in Bagerhat and Jhalokati. High-low outliers (districts with high case concentrations surrounded by low case concentrations) were observed in Kishoreganj and Rangpur. In 2022, Gopalganj emerged as a low-high outlier. Interestingly, no spatial outliers were identified in 2022 (Fig 7).

## Discussion

Our analysis of dengue incidence in Bangladesh from 2019 to 2023 (excluding 2020) revealed distinct spatial patterns. Southeast and central regions consistently exhibited high incidence with districts like Barisal and Faridpur emerging persistent hotspots. Conversely, the north and northeast remained coldspots with minimal dengue incidence. Spatial autocorrelation analysis confirmed significant clustering of high or low incidence districts, suggesting non-random distribution of the disease across Bangladesh.

Our findings align with previous research highlighting Dhaka’s historical role as a dengue epicenter. However, surrounding districts with lower incidence rates prevented its classification as a significant hotspot itself in this study. Recognizing established hotspots like Bagerhat, Barisal, Faridpur, and Jhalokati is crucial for targeted interventions, a notion supported by multiple studies [5,13,16,17]. These regions are particularly vulnerable due to their location in the Ganges-Brahmaputra-Meghna delta, where they are exposed to potential climate-induced changes. These changes include hazards like floods, prolonged rainy seasons, droughts, and the effects of El Niño and La Niña, all of which can impact mosquito distribution [12,30–36]. Additionally, intra-urban transportation networks within these hotspots might also influence disease spread [37]. This spatial understanding empowers policymakers to develop targeted strategies for combating dengue transmission. Such strategies could include prioritizing resources like insecticide spraying in high-risk areas, launching public awareness campaigns in hotspots, and implementing policies promoting mosquito control and proper waste management. Ultimately, these insights can guide the development of effective control measures to combat dengue transmission.

Conversely, the northern and northeastern regions consistently exhibited lower case concentrations, establishing them as coldspots for dengue transmission. Environmental or climatic factors in these regions, such as lower temperatures, reduced humidity, or a scarcity of breeding sites in these regions might be less conducive to dengue transmission. This aligns with prior research emphasizing targeted strategies for low incidence areas [16]. Investigating the specific factors that contribute to the low rates in coldspots offers valuable insight that can inform the development of more effective prevention and control efforts across the country.

Intriguingly, our study also revealed an earlier peak in the geographical spread of dengue cases in 2023 dengue outbreak, with the peak occurring three months earlier than in 2022. This earlier peak may be associated with changes in temperature and rainfall patterns affecting mosquito breeding. In addition to this rapid urbanization can also create new breeding sites [5]. However, it is important to consider limitations such as potential changes in data collection points (e.g., greater number of health care center) that could influence observed trends.

Moreover, dynamic shifts in rural landscapes and evolving human- environment interactions contribute to the heterogeneity of dengue transmission across diverse rural and urban settings [14,16]. The findings of this study, particularly the shifting epicenter (e.g., from Dhaka to Manikganj) and temporal variations (earlier peak in 2023), underscore the dynamic nature of dengue in Bangladesh. While data on rural landscape changes within hotspots is limited, rapid unplanned urbanization in these areas might be a contributed factor. This unplanned growth might create ideal breeding grounds for mosquitoes, similar to those observed during rural-to-urban transitions. These insights underscore the importance of strategically allocating resources, adapting vector control measures, and directing public awareness campaigns in hotspots to inform effective control strategies for endemic regions.

Out study identified a concerning upward trend in the dengue case fatality rate (CFR) from 2019 to 2023. This rise in CFR may be attributable to several factors including an increase in secondary infections, changes in circulating serotypes, and potential limitations within the healthcare system during outbreaks [5,15,38]. Strengthening healthcare infrastructure, especially at the district level, alongside public awareness campaigns promoting early detection of dengue symptoms, could be crucial for mitigating CFR in future outbreaks.

The study acknowledges limitations inherent to the existing dengue surveillance system in Bangladesh which captures data from only approximately 5% of healthcare facilities nationwide [15]. Additionally, the exclusion of 2020 data and reliance solely on case and population data restrict our ability to fully elucidate spatiotemporal trends. Future research should incorporate additional data sources, such as climate and environmental factors, and conduct fine-scale sub-district level analysis for a more comprehensive understanding. Exploring the factors contributing to hotspots, along with the underlying dynamics of transmission is crucial for designing targeted interventions and mitigating future outbreaks.

In conclusion, this study sheds light on the dynamic spatiotemporal patterns of dengue in Bangladesh. The emergence of Manikganj with high dengue incidence, alongside consistently high caseloads in urban centers like Dhaka, underscores the evolving nature of the disease in Bangladesh. These findings, coupled with observed temporal patterns and spatial clustering underscore the critical need for adaptable public health strategies. The identification of persistent and emerging hotspots and coldspots provides a crucial foundation for implementing targeted interventions and flexible public health measures to effectively combat the evolving dynamics of dengue transmission in Bangladesh.
